# Expression Profiling Reveals Novel Hypoxic Biomarkers in Peripheral Blood of Adult Mice Exposed to Chronic Hypoxia

**DOI:** 10.1371/journal.pone.0037497

**Published:** 2012-05-22

**Authors:** Matias Mosqueira, Gabriel Willmann, Ulrike Zeiger, Tejvir S. Khurana

**Affiliations:** 1 Department of Physiology and Pennsylvania Muscle Institute, University of Pennsylvania School of Medicine, Philadelphia, Pennsylvania, United States of America; 2 Medical Biophysics Group, Institute of Physiology and Pathophysiology, Heidelberg University, Heidelberg, Germany; 3 Department of Vegetative Physiology, University of Cologne, Cologne, Germany; Kaohsiung Chang Gung Memorial Hospital, Taiwan

## Abstract

Hypoxia induces a myriad of changes including an increase in hematocrit due to erythropoietin (EPO) mediated erythropoiesis. While hypoxia is of importance physiologically and clinically, lacunae exist in our knowledge of the systemic and temporal changes in gene expression occurring in blood during the exposure and recovery from hypoxia. To identify these changes expression profiling was conducted on blood obtained from cohorts of C57Bl-10 wild type mice that were maintained at normoxia (NX), exposed for two weeks to normobaric chronic hypoxia (CH) or two weeks of CH followed by two weeks of normoxic recovery (REC). Using stringent bioinformatic cut-offs (0% FDR, 2 fold change cut-off), 230 genes were identified and separated into four distinct temporal categories. Class I) contained 1 transcript up-regulated in both CH and REC; Class II) contained 202 transcripts up-regulated in CH but down-regulated after REC; Class III) contained 9 transcripts down-regulated both in CH and REC; Class IV) contained 18 transcripts down-regulated after CH exposure but up-regulated after REC. Profiling was independently validated and extended by analyzing expression levels of selected genes as novel biomarkers from our profile (e.g. spectrin alpha-1, ubiquitin domain family-1 and pyrroline-5-carboxylate reductase-1) by performing qPCR at 7 different time points during CH and REC. Our identification and characterization of these genes define transcriptome level changes occurring during chronic hypoxia and normoxic recovery as well as novel blood biomarkers that may be useful in monitoring a variety of physiological and pathological conditions associated with hypoxia.

## Introduction

Since the pioneering studies of Paul Bert, Denis Jourdanet and Francois-Gilbert Viault conducted in late 1800, it is well known that hypoxia, due to reduced oxygen tension noted at high altitude, is a primary physiological stimulus for erythrocyte production in both animals and humans [1], [2]. Early in the 20^th^ century, Carnot and De Flandre suggested the existence of a humoral agent that responds to hypoxia and induces erythropoiesis [3]. Later, several experiments demonstrated that plasma contains the humoral agent erythropoietin (EPO) capable of inducing hematopoiesis during hypoxic and anemic conditions [4]. In 1977, Miyake *et al.* purified EPO from human urine samples [5], which facilitated its cloning and consequently the production of recombinant EPO used nowadays to treat certain types of anemia [6]. Under hypoxic conditions, kidney and liver are the major sites of EPO production and bone marrow is the major target. EPO acts synergistically with granulocyte-macrophage colony-stimulating factor, stem cell factor, and interleukins 3, 4 and 9 as well as insulin growth factor-1 in the later stages of erythropoiesis. Along with EPO, other cytokines and growth factors induce proliferation, maturation and survival first in the burst forming unit-erythroid and then in the colony forming unit-erythroid. The final result is an increase of reticulocytes which can be measured in the peripheral blood stream, and consequently an increase in the number of mature erythrocytes and hematocrit [6].

The molecular mechanism of hypoxia-induced over-expression of EPO remained elusive until 1995 when Wang & Semenza [7] described hypoxia inducible factor (HIF). HIF belongs to the PAS family of transcription factors and consists of an oxygen-sensitive α-subunit and an oxygen insensitive β-subunit. Three α-subunits have been described for HIF (−1α, −2α, and −3α), which under normoxia are rapidly targeted by the von Hippel-Lindau tumor suppressor or prolyl-hydroxylation and subsequently degraded. Under hypoxic conditions, HIF-1α and HIF-2α stabilize and heterodimerize with HIF-β to form HIF and thus initiate a multistep pathway of activation that includes nuclear translocation and recruitment of transcriptional co-activators, to act as a transcriptional regulator at the hypoxia-response element (HRE) binding site for the hypoxia-sensitive enhancer located in the 3′ Untranslated Region (UTR) of the gene [8]. The action of HIF is limited to short periods of hypoxia and its stabilization is tissue-dependent. Mice exposed to a FiO_2_ of 6% present different kinetics of HIF stabilization for brain, kidney and liver. In the brain, the peak of stabilization occurs after 4–5 hours of hypoxia, returning to normoxic levels after 9–12 hours of hypoxia. In kidney and liver, the peak of HIF occurs after 1–2 hours, returning to normoxic levels after 3 hours exposure to hypoxia [9]. HIF mRNA levels from brain, kidney and liver exposed to hypoxic conditions remain unchanged compared to normoxic conditions [9], supporting the hypothesis of post-translational degradation. These results suggest that *in vivo* HIF is important to regulate acute systemic and local responses, such as regional changes in blood supply, but may not play a major role in the acclimatization to chronic hypoxia [9], [10].

The hypoxia-induced gene expression mechanism is present in almost all types of cells inducing metabolic and structural changes according to the tissue type, tissue requirements and intensity of the hypoxic challenge. More than 80 possible HIF-target genes from several pathways have been described [11]. Although there is an extensive list of genes that respond to hypoxia, only the EPO receptor, CD36 (marker for early erythroid cells) and γ-globin [12] have been suggested and used as biomarkers for hypoxia-induced erythropoiesis in blood cells. While obtaining whole blood sample is an easy and fast technique, the lack of additional biomarkers makes it difficult to differentiate between acute and chronic hypoxia exposure as well as from normoxic recovery stages. To address this, we used microarray analysis to identify changes in gene expression occurring in the peripheral blood cells of mice exposed to chronic hypoxia and normoxic recovery to study their gene expression levels. We independently validated the transcriptome changes by qPCR and have identified three novel blood biomarkers based on these transcriptome levels changes.

## Methods

### Animals

**Figure 1:**
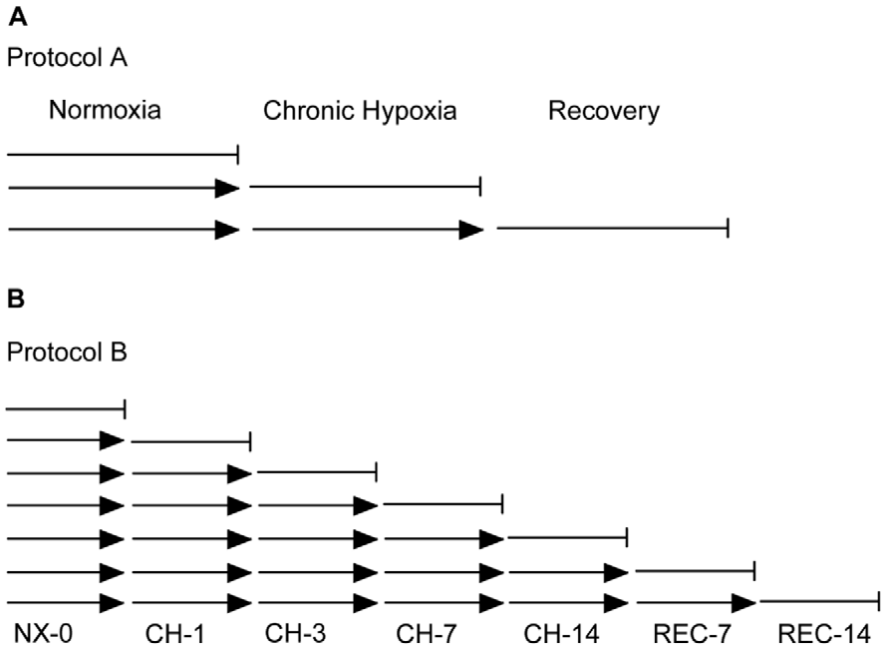
Protocols for molecular signature and validation. A. Animals were divided into three groups of 5 mice each. Group 1 was exposed to normoxia (NX) for two weeks; group 2 exposed to two weeks of NX and then two weeks of chronic hypoxia (CH); group 3 was exposed to two weeks of NX, followed by two weeks of CH and another two weeks of normoxic recovery (REC). B. Protocol 2 was designed to test the expression levels of differentially expressed genes from the microarray analysis. Each day point corresponds to a group of five mice exposed to normoxia (NX-0), chronic hypoxia (CH-1, CH-3, CH-7 and CH-14) and recovery (REC-7 and REC-14).

All animal experiments were approved by the Institutional Animal Care and Use Committee (permit number 80888) of the University of Pennsylvania School of Medicine. Adult normal (C57Bl/10) mice aged 3 months were obtained from Jackson Laboratory (Jackson Laboratory, Bar Harbor, Maine, USA). Hypoxic exposure was undertaken in a specially designed, hermetically closed hypoxic chamber using the Pegas 4000 MF gas mixing system (Columbus Instruments, Ohio USA). The oxygen level was gradually decreased from 21% to 8% (Table 1). The weights of the animals were monitored daily and food and water were offered *ad libitum*. In Protocol 1 (Figure 1A), mice were randomly divided into three groups for gene expression profiling: normoxic (NX), chronic hypoxic (CH) and recovery (REC); each group consisted of 4 animals. The groups were exposed for 14 days to NX (NX; group 1), 14 days to CH (CH; group 2) and 14 days to CH followed by 14 days of normoxia (REC; group 3). In Protocol 2 (Figure 1B) five mice were randomly selected from each group to one of the seven day points (NX-0, CH-1, CH-3, CH-7, CH-14, REC-7 and REC-14). All animals used in both protocols 1 and 2 presented similar initial body weight.

**Table 1:** Protocol for CH and REC.

Day	FiO_2_ (%)	Microarray	Biomarker
**NX-0**	21	•	•
**CH-1**	15		•
CH-2	13		
**CH-3**	12		•
CH-4	11		
CH-5	10		
CH-6	9		
**CH-7**	8		•
CH-8	10		
CH-9	9		
CH-10	9		
CH-11	9		
CH-12	8		
CH-13	8		
**CH-14**	8	•	•
REC-1	21		
REC- 2	21		
REC-3	21		
REC-4	21		
REC-5	21		
REC-6	21		
**REC-7**	21		•
REC-8	21		
REC-9	21		
REC-10	21		
REC-11	21		
REC-12	21		
REC-13	21		
**REC-14**	21	•	•

Protocol used for Microarray profile (Protocol 1) and Biomarker (Protocol 2) experiments. The • indicates the peripheral blood harvest from 5 different animals. The fraction of inspiration (FiO_2_) was changed every 24 hours.

### RNA Isolation for Microarray Hybridization

Intravenous blood was collected and approximately 50 µL of blood was used for hematocrit measurement and the rest (c. 750 µL) was saved in RNALater (Qiagen, Valencia, CA, USA) for further analysis. Total RNA was isolated from blood samples using Tri-Reagent (Ambion, Austin, TX, USA) as described by the manufacturer. Isolated total RNA was cleaned up by RNeasy mini kit (Qiagen, Valencia, CA, USA) according to the manufacturer’s instructions. The purity and concentration of total RNA were determined by measurement of absorbance at 260 and 280 nm using a Nanodrop ND-1000 spectrophotometer (NanoDrop Technologies, Wilmington, DE, USA). To satisfy our purity criteria, we required that all RNA.

samples used in our experiments had a 260/280 ratio between 1.8 and 2.1. RNA integrity was monitored by determining the28S/18S ratio using an Agilent 2100 Bioanalyzer (Agilent Technologies Inc., Santa Clara, CA, USA).

### Linear Amplification and cRNA Labeling for Microarray Hybridization

Three microliter total RNA was used for each sample to obtain linearly amplified labeled cRNA by using GeneChip® One-Cycle Target Labeling kit (Affymetrix, Santa Clara, CA, USA) as described by the manufacturer. Briefly; total RNA was used to generate double-stranded cDNA with the T7-oligo (dT) primer. This double-stranded cDNA was used for in vitro transcription and biotin labeling steps. Labeled cRNA yield and purity were determined by measuring the absorbance at 260 and 280 nm. All the cRNA 260/280 ratios were between 1.9 and 2.1. Quality control of the labeled cRNA products was assessed by performing gel electrophoresis using 1 µg labeled cRNA on a 2% agarose gel.

### Fragmentation and Microarray Hybridization

Fifteen micrograms labeled cRNA extracted from NX, CH and REC samples were fragmented and 10 µg were hybridized to Affymetrix® Mouse 430 ver. 2.0 GeneChip arrays for 18–24 hrs (Affymetrix Inc.). Each microarray was washed and stained with streptavidin–phycoerythrin and scanned at a 6-µm resolution with an Agilent model G2500A GeneArray scanner. A visual quality control measurement was performed to ensure proper hybridization after each chip was scanned. Other quality control parameters such as scaling factors used to normalize the chips, average background, and noise were also evaluated. In addition, raw intensities for each probe set were stored in electronic formats by the GeneChip Operating System version 1.1 (GCOS1.1, Affymetrix Inc.). Primary data is accessible in the GEO database (Accession number GSE 17728).

Data was normalized using the GC-RMA algorithm in Partek Genome Suite (Partek Incorporate, St Louis, MO). Differentially expressed transcripts were identified at the level of two fold change cut-off and 0% FDR. Differentially expressed transcripts were clustered using the functional annotation clustering tool of the Database for Annotation, Visualization and Integrated Discovery (DAVID) program [13], [14].

### Validation of Gene Expression by qPCR

Real time quantitative polymerase chain reaction (qPCR) was used to measure the relative expression level of mRNA of four genes (spectrin alpha-1, ubiquitin domain family-1, pyrroline-5-carboxylate reductase-1 and CD-36) in five independent blood samples harvested from animals exposed to Protocol 2 (Figure 1B). Briefly, 100 ng of RNA was extracted from blood, reversed transcribed in 20 µL final reaction using random hexamers and the SuperScript First-Strand Synthesis according to the manufacturer (Invitrogen, Carlsbad, CA). Two ng of cDNA was amplified in 20 µL of reaction mixtures containing 1 pmole of forward and reverse primers each, 10 µL of 2× TaqMan universal PCR master mix and 0.25 M probes (Table 2). The amplification was performed in a 7900HT Sequence Detection System (ABI). Samples were run in triplicates.

**Table 2:** List of genes used for validation of gene expression.

Name	TAQMAN® Gene Expression Assay ID	REFSEQ
**CD36**	Mm00432403_m1	NM_001159557.1
**Spna1**	Mm00501882_m1	NM_011465.4
**Ubfd1**	Mm01165153_m1	NM_138589.2
**Pycr1**	Mm00522674_m1	NM_144795.3
**Actb**	Mm00607939_s1	NM_007393.3

Name, TAQMAN catalog number from Applied Biosystems® and REFSEQ from each gene used on validation of microarray study. The last gene is the housekeeping gene used.

### Hematocrit Measurement and Reticulocyte Counting in Peripheral Blood

Hematocrit was measured using blood collected in heparinized micro-hematocrit capillary tubes (Fisher, Pittsburgh, PA) and centrifuged for two minutes using an International micro-capillary centrifuge model MB. Two measurements were made for each animal and the average value was calculated. Reticulocyte counts were performed on thick blood films made using 300 µL of peripheral blood collected in a syringe with 30 µL EDTA 0.5 M as anti-coagulant. The wedge method was used for making the blood film [15]. Brilliant Cresyl blue supravital staining was performed according to manufacturer’s protocols (ENG Scientific, Clifton, NJ; Catalog number 5111). The number of erythrocytes and reticulocytes were manually counted using a microscope at 100× magnification and reticulocyte percentage was calculated [15].

## Results

### Expression Profile

**Figure 2:**
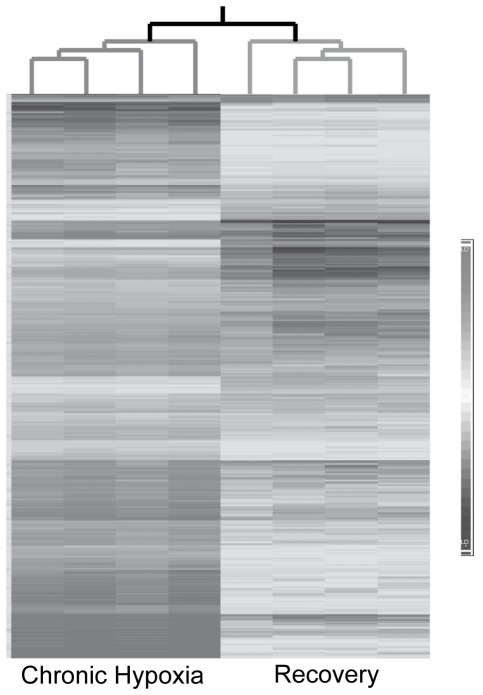
Heatmap and dendrogram representations of all 512 transcripts that were differentially expressed in CH and REC. Each column represents one sample (chip) and each row a differentially expressed transcript. The intensity of the transcript expression is indicated by the gray shaded bar at the right side of the figure. The four chronic hypoxia (CH) and recovery (REC) GeneChip data sets cluster into two distinct groups based on correlation of gene expression pattern. The dendrogram branches lengths for CH and REC sub-trees seen at the top are based on normalized raw data of all transcripts and quantitatively demonstrate that the four samples are closely related to each other. Each horizontal bar represents one probe set, and the intensity of gray color of the bar determines the degree of expression.

To identify and define gene expression changes that occur in peripheral blood during hypoxia and normoxic recovery, expression profiling was conducted on blood obtained from cohorts of C57Bl-10 wild type mice. The animals were maintained either at normoxia (NX), exposed for two weeks to normobaric chronic hypoxia (CH) or two weeks of CH followed by two weeks normoxic recovery (REC) as described in Protocol 1 (Figure 1). Out of the 45,101 probe sets present on the array, 512 probe sets (representing equal numbers of transcripts) showed significant (p<0.05 after multiple testing correction) differential expression across the three experimental conditions (NX, CH, REC) upon the application of ANOVA, a 0% FDR and a twofold change cut-off. Out of these probe sets, 230 transcripts showed a significant differential expression in both CH and REC conditions (Supplemental Table S1). Heatmap analysis (Figure 2) demonstrates the relative changes in transcript expression of two weeks of CH exposure and two weeks of CH followed by two weeks of REC (Protocol 1, Figure 1A). These results were compared to two weeks of exposure to NX (control conditions). The differential clustering of the samples, visually observed in the heatmap was confirmed by principal component analysis (PCA). The CH samples are more dispersedly distributed, but still significantly different from control and recovery samples. PCA axes 1, 2 and 3 were 49.7%, 12.9% and 9.05%, respectively.

**Figure 3:**
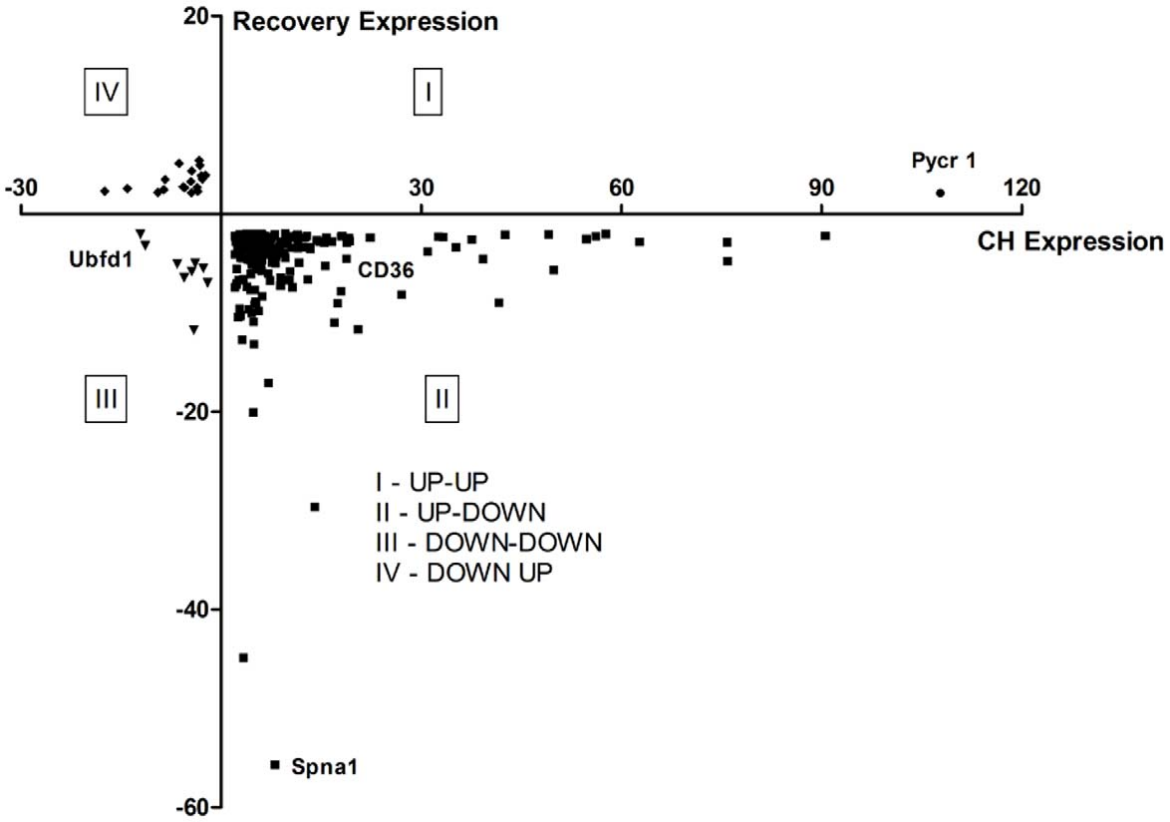
Fold change relationship of differential gene expression. The 230 differentially expressed genes were divided into four different sets according to their expression over time. The abscissa represents the fold change of the CH transcript expression profile, and the ordinate represents the fold change of the REC transcript expression profile. The first quadrant (I) shows one gene (circle) up-regulated in both CH and REC. The second quadrant (II) shows the 202 genes (square) up-regulated during CH and down-regulated after REC. The third quadrant (III) shows the 9 genes (triangle) down-regulated in both CH and REC conditions. The fourth quadrant (IV) shows 18 genes (diamond) which are down-regulated during CH and up-regulated after REC.

The 230 transcripts were classified into four different categories according to their expression in CH and during REC (Figure 3). The first quadrant (I) contained one transcript that was up-regulated in both CH and REC conditions; the second quadrant (II) contained 202 genes up-regulated in CH followed by down-regulated expression after two weeks of REC; the third quadrant (III) contained 9 transcripts that were down-regulated during both CH and REC; the fourth quadrant (IV) showed 18 transcripts that were down-regulated in CH exposure followed by up-regulated expression after two weeks of REC. Only the genes contained in the second quadrant reached the minimum number to enable meaningful functional annotation clustering analysis using DAVID [13], [14]. The significantly enriched clusters revealed processes related to cellular catabolic processes, followed by metabolic processes and hematopoiesis (Supplemental Table S2).

### Effects of CH and REC on Body Weight, Hematocrit and Reticulocytes

**Figure 4:**
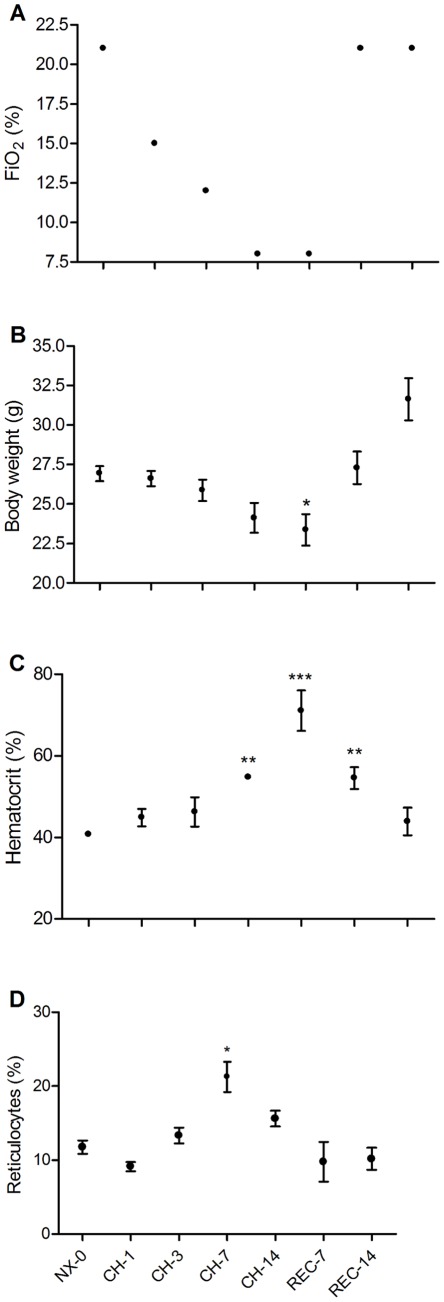
Effect of CH and REC on body weight, hematocrit and reticulocytes. A. FIO_2_ was reduced from 21% to 8% (CH) over a two week period, to return to 21% for two weeks (REC). B. Body weight (BW) was reduced during CH and rapidly returned to a normal level during REC. C. Hematocrit increased during CH and returned to normal levels after 14 days of REC. D. Relative amount of reticulocytes increased until day 7 of CH and then returned to normoxic levels (NX) during REC. Mean ± SEM; n = 5 per day point. * p>0.05; ** p>0.01; *** p>0.001, relative to NX-0.

In order to study more precisely the effects of chronic hypoxia and recovery during normoxia over time and to correlate these with changes in gene expression, we designed a second protocol, in which we weighed the animals and harvested peripheral blood samples at 7 different day points (NX-0, CH-1, CH-3, CH-7, CH-14, REC-7 and REC-14; Figure 1B). Figure 4A shows the decreasing FiO_2_ levels during protocol 2, namely CH from 21% down to 8% and then the recovery at 21%. The average body weight (BW) decreased during CH exposure (Figure 4B), reaching the minimum at CH-14 (Table 3). However, the BW rapidly recovered from CH returning to similar levels as NX-0 at REC-7. Hematocrit significantly increased at CH-7, reaching its maximum level at CH-14 (Table 3). After return to normoxia, the hematocrit remained significantly above NX-0 levels until REC-7 (Figure 4C), returning to a normal level on REC-14. The relative amount of reticulocytes significantly increased at CH-7 (Table 3), and correlated to the first significant rise of the hematocrit on CH-7 rather than at day 14 of CH when the hematocrit peaked.

**Table 3:** Body weight and hematological response to CH and recovery exposures.

Day	FiO_2_ (%)	BW (g)	Hematocrit (%)	Reticulocytes (%)
**NX-0**	21	26.94±0.21	40.80±0.20	11.76±0.90
**CH-1**	15	26.62±0.22	44.90±0.94	9.13±0.62
**CH-3**	13	25.88±0.30	46.30±1.61	13.33±1.05
**CH-7**	8	24.12±0.42	54.80±0.12**	21.26±2.05*
**CH-14**	8	23.38±0.44 *	71.10±2.20***	15.62±1.07
**REC-7**	21	27.30±0.46	54.58±1.19**	9.77±2.68
**REC-14**	21	31.64±0.59	43.94±1.51	10.17±1.49

Values in Mean ± SEM. Significant differences compared to day 0.

* p<0.05.

** p<0.01.

*** p<0.001.

### Validation of Gene Expression by qPCR and Characterization of Potential Biomarkers for CH and REC

**Figure 5:**
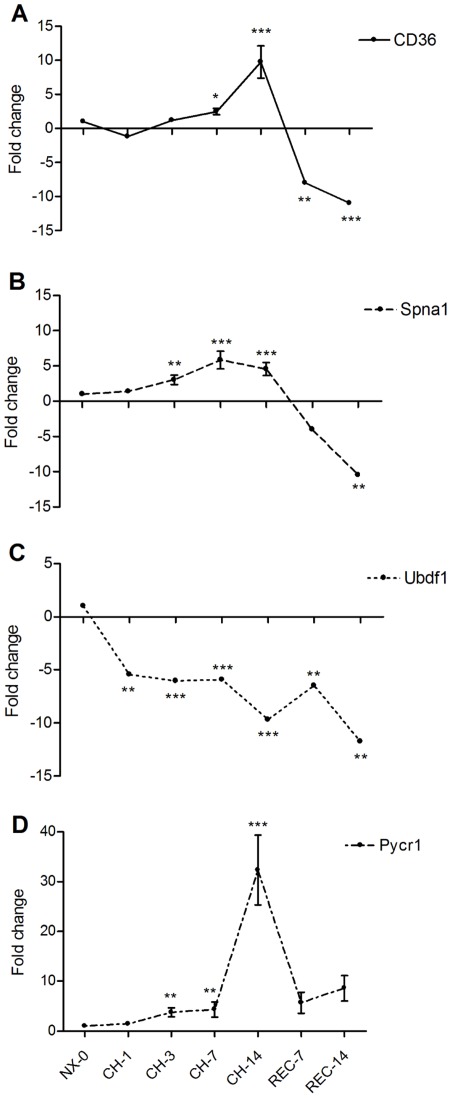
Expression of whole peripheral blood biomarkers exposed to chronic hypoxia. Real time qPCR was normalized using the expression of β-actin as a housekeeping control. A. CD36 as a marker for erythropoiesis increased the expression level during chronic hypoxia (CH), but decreased below normoxic levels during REC. B. Spna1 presented a similar pattern as CD36, by increasing its gene expression level during CH and decreasing it below normoxic levels after REC. C. Ubfd1 remained down-regulated during the entire protocol of CH and REC. D. CH up-regulated Pycr1 which remained up-regulated for 14 days of REC. Mean ± SEM; n = 5 per day point. * p>0.05; ** p>0.01; *** p>0.001 Relative to NX-0.

In order to independently validate our profiling as well as to extend our studies toward identifying novel biomarkers in peripheral blood from mice challenged to CH and REC, we selected three candidate genes detected in our microarray study: spectrin α-1 (Spna1), ubiquitin family domain containing 1 (Ubfd1) and pyrroline-5-carboxylate reductase 1 (Pycr1). We performed qPCR analyses on independent samples taken from different groups of animals at day NX-0, CH-1, CH-3, CH-7 and CH-14 during hypoxia exposure and at REC-7 and REC-14 (Protocol 2, Figure 1B). To correlate the expression of our set of genes with the hematopoietic response to hypoxia, we also studied the gene expression level of CD36, a known marker for erythroid progenitors [12]. The fold changes were compared to levels at day 0 (Table 4). As shown in Figure 5, CD36 levels significantly increased its expression level only at CH-7, reaching its maximum at CH-14; once returned to normoxic conditions the expression of CD36 decreased significantly and remained down-regulated at REC-14 (Figure 5A). The expression of Spna1 increased significantly one day earlier than CD36, i.e. at CH-3 and remained up-regulated during CH exposure; nevertheless, during REC the expression of Spna1 decreased, reaching the lowest level at REC-14 (Figure 5B). As predicted by the microarray profile, the expression of Ubfd1 was significantly below normoxic levels during the entire period of CH exposure and remained down-regulated over the 2 weeks of REC (Figure 5C). Finally, consistent with the microarray predictions, Pycr1 expression showed an early response with a significant increase at CH-3 and remained up-regulated throughout CH exposure until REC-14 (Figure 5D).

**Table 4:** Gene expression response to CH and REC exposure.

Day	CD36	Spna1	Ubdf1	Pycr1
**CH-1**	−1.18±0.13	1.39±0.29	−5.44±0.04 (0.002)	1.45±0.37
**CH-3**	1.20±0.25	3.04±0.67 (0.015)	−6.04±0.04 (0.001)	3.76±0.90 (0.008)
**CH-7**	2.46±0.48 (0.012)	5.84±1.28 (0.001)	−5.91±0.04 (0.001)	4.36±1.54 (0.002)
**CH-14**	9.76±2.38 (0.001)	4.56±0.93 (0.001)	−9.69±0.10 (0.001)	32.31±7.00 (0.001)
**REC-7**	−8.00±0.04 (0.001)	−4.02±0.09	−6.47±0.05 (0.001)	5.71±2.11
**REC-14**	−10.97±0.02 (0.002)	−10.46±0.03 (0.033)	−11.75±0.03 (0.018)	8.62±2.54

Fold changes values compared to Day 0 (NX-0). Values are in Mean ± SEM. Significant *p*-values are shown in brackets.

## Discussion

Our goal in this study was to identify the systemic and temporal changes in gene expression occurring in blood during the exposure and recovery from hypoxia to identify novel genes to be used as possible reference genes (biomarkers) detectable by qPCR in small samples of peripheral blood. Since all blood cells were used rather than sorted by cell types, we cannot attribute contribution of gene changes to specific peripheral blood cell type or types, and thus discuss the changes in gene expression and analyses as an effect of CH present in the whole peripheral blood.

Out of 230 differentially regulated transcripts found to be differentially regulated, we did not identify any differentially expressed transcripts from the list of 86 HIF-regulated genes that have been detected in a stringent meta-analysis across 6 cell types [11]. However, we did identify 5 genes differentially expressed in liver from mice exposed to CH [10] (solute carrier family 16, B-cell translocation gene 3, testis-specific protein; Y-encoded-like 1, CD36 antigen and preimplantation protein 4). Another comparison of our differentially regulated genes with a set of genes derived from bone marrow of mice exposed to intermittent hypoxia also revealed only 15 genes to be in common as shown in Supplemental Table S3 [16]. These comparisons show that HIF may not directly regulate the expression of our 230 transcripts during CH and support the evidence that while HIF is important during the acute phase response during hypoxia to regulate local and systemic mechanisms that underpin oxygen sensing [9], [10], it may not play a major role in the chronic phase of acclimatization to CH. However, comparisons with 11 other studies who have studied the effects of hypoxia across different species and tissues using differing experimental protocols for hypoxia exposure revealed no genes in common with our expression profile suggesting its specificity to tissue type and condition studied [17], [18], [19], [20], [21], [22], [23], [24], [25], [26], [27].

As a systemic response for acclimatization to CH, we observed a significant loss of BW over the period of CH exposure, reaching its lowest level at the end of the CH protocol (CH-14), confirming previous reports [28]. However, during recovery, the BW rapidly recovered to similar levels as before CH exposure. Hypoxia-induced BW loss has been extensively reported [28], [29], [30], [31], [32]. The mechanisms involving BW loss are thought to be mediated mainly by carbohydrate and lipid metabolisms. The glucose metabolism has been extensively studied and is considered a major energy pathway involved in the response to hypoxia [8]. Subjects lost weight and did not increase their blood sugar level despite drinking 100 grams of glucose after a 17 days-expedition to the Chilean Andes exposed to high altitude (5340 MASL) [33]. CH induces the expression of glucose transporter (GLUT) types 1 and 4 in rat cardiac and skeletal muscles [29], [32] and GLUT1 in the brain blood barrier [30], suggesting that the lower plasma glucose induced by CH leads to higher expression and activity of GLUT in several tissues. Finally, hypoxia inhibits liver gluconeogenesis, subsequent to a reduction in phosphoenolpyruvate carboxykinase activity [34]. Together, these results suggest that the loss of BW during CH exposure may be attributed to increased carbohydrate catabolism.

A novel mechanism for BW regulation during hypoxia may involve the leptin pathway, which is a regulator of BW and lipid metabolism during hypoxia. Leptin is an obese (ob) gene product and its mature form contains a 167 amino-acid peptide hormone [35]. It is produced in adipose tissue [36], placenta [37], stomach [38], muscle tissue [39], and cerebral adenohypophysis and hypothalamus [40]. The action of leptin is mediated by 6 different isoforms of the receptor present in the hypothalamus [41], [42], regulating BW by suppressing food intake and increasing the energy expenditure, glucose and fatty acid metabolism, hematopoiesis and immune system [43], [44], [45], [46]. The ob gene contains a HRE site that can be bound by HIF in its 5′-untranslated region inducing up-regulation of leptin expression under hypoxic challenges [47], [48]. Recently, Baze *et al.* showed that chronic exposure to hypobaric hypoxia (4500 MASL) increases the leptin receptor expression in liver samples [10]. In concordance with those results, rats exposed during 90 days at 5500 MASL showed an increase of lipid metabolism in both liver and skeletal muscle [49]. These results suggest that fatty acid catabolism, hematopoiesis and energy expenditure are important factors for acclimatization to hypoxia and that the leptin pathway may play a profound role as a part of the mechanism for BW regulation noted during CH acclimatization.

We observed that CH and REC treatments had significant effects on classical peripheral blood parameters. The hematocrit level increased from 40% at NX to 54% and 74% at CH-7 and CH-14, respectively. The levels of hematocrit from CH-14 only returned to similar levels of NX-0 normal levels after 14 days of REC. Interestingly, the hematocrit at REC-7 presented similar levels as CH-7, suggesting that the turnover of erythrocytes is similar during the waxing and waning of hematopoeisis during CH and REC. It is well established that hypoxia induces EPO expression under either physiological or pathophysiological conditions, resulting in an increase of reticulocyte, hematocrit and hemoglobin concentration [1], [50]. While the relative numbers of reticulocytes were noted to be increased at CH-3 compared to NX-0, the increase became significant at CH-7, but did not continue to increase after 14 days of CH compared to CH-7. Rather, the expression level decreased, but still remained above normoxic level. The percentage of reticulocytes showed similar levels as at NX-0 once returned to REC. Our results are in concordance with studies performed in humans exposed to either intermittent or continuous CH (7 days, FiO_2_ 15.4%) which reported an increase of the relative amount of reticulocytes on the 5^th^ and 7^th^ day [51]. The hypoxia-induced increase in reticulocytes is also known to occur during prolonged hypoxic exposure. After a 21-day expedition to the Andes with continuous exposure above 4000 MASL (maximum of 6768 MASL), subjects submitted to four hours in a hypobaric chamber (4500 MASL, 443 mmHg) showed a new increase in the number of reticulocytes, as well as in the percentage of red blood cells [31]. This result shows the capacity of colony forming unit-erythroid to response to new hypoxic challenge and continue to form new reticulocytes. [31]. This hypoxia-mediated reticulocytosis phenomenon has also been reported in animals, with a slightly different time-course response. A study using rats exposed to a hypobaric hypoxia protocol (21 days at 0.5 atm; PB 380 mm Hg) showed an increase in the relative number of reticulocytes on day 3, reaching the maximum on day 7, before returning to normal levels on day 14. However, the reticulocytes reached a new peak on day 21 of the protocol [52]. These results suggest a negative feedback on the hypoxia-induced reticulocytes formation, which reaches its maximum after 5–7 days of hypoxia before decreasing to normal levels, as we have described here.

One of the major aims of our study was to analyze the expression profile to identify and validate novel genes that could be used as reference genes (biomarkers) of CH and REC. Towards this goal we studied the expression levels of Spna1, Ubfd1 and Pycr1 as well as the previously characterized CD36 gene expression in detail. The pattern of differential expression of Spna1 paralleled changes in hematocrit during CH and REC, while Ubdf1 and Pycr1 genes were down- and up-regulated respectively, during the CH and REC periods. CD36 is a 88 KDa cell surface protein that belongs to an evolutionary conserved Class B scavenger receptors family [53], [54]. The expression of CD36 in erythroid cells is limited to reticulocytes, absent in mature erythrocytes [55], [56] and has been used as a marker for early erythroid cells [12]. Acute hypoxia up-regulates CD36 in intrapulmonary arteries via activation of HRE mediated by HIF [57], [58]. In our microarray analysis, CD36 was up-regulated in mice peripheral blood during CH (fold-change 16.65) and then down-regulated during REC (fold-change -2.85). This pattern of expression was confirmed by qPCR in our extended time-point protocol 2, showing a significant increase in CD36 expression already after day 2 of CH and a marked down-regulation upon return to normoxia. The significant increase partially overlapped with the increased relative amount of reticulocytes on CH-7. This difference may be explained by the proliferation of reticulocytes induced by hypoxia [59]. Nevertheless, the expression of CD36 was completely suppressed by REC, suggesting that reticulocyte proliferation activity was also reduced during REC.

Spna1 is a 280 KDa subunit of the spectrin cytoskeletal protein [60], [61], [62], [63]. Spectrin is a major constituent of red blood cell membranes and responsible for resistance to mechanical stress, cellular form and anchors for other proteins such as hemoglobin receptors and channels [64], [65]. Our results suggest that in whole blood samples, Spna1 expression is increased in response to CH. However its expression level declined during REC reaching NX levels, paralleling changes in hematocrit.

Ubfd1 is a novel polyubiquitin binding protein [66]. Although hypoxia-induced changes in expression have not previously been described, analysis of primary data (GEO number GDS1807) in a study using a human lymphocyte cell line exposed to FiO_2_ 0.1% for 29 hours demonstrated down-regulation of two transcripts (224878_at and 20568_at) of this gene [67]. These data are in agreement with our demonstration that Ubfd1 expression is sensitive to CH and remained significantly down-regulated from the beginning of the hypoxic exposure until several days after the REC.

Pycr1 is a mitochondrial enzyme that converts pyrroline-5-carboxilic acid into proline in a NAD(P)H-dependent reaction [68]. Pycr1 generates an oxidizing potential to stimulate the metabolism of glucose through the hexose monophosphate-pentose pathway and the Embden-Meyerhof pathway, an important pathway in erythrocytes [69]. The increase of Pycr1 expression during CH suggests an increase of erythrocyte metabolism and glucose consumption in order to increase the amount of NAD(P)^+^, not limiting the production of ATP via the anaerobic pathway of glycolysis. In astrocytes, 24 hours of hypoxia up-regulates Pycr1 expression and has been shown to be mediated by p53 [70]. In our profile, Pycr1 expression increased upon CH challenge, increased its expression further during CH and remained above the normoxic expression level even after 14 days of REC.

In conclusion, we describe the molecular signature for whole peripheral blood samples from mice challenged to CH and REC. The expression profile of genes separable into four distinct temporal categories (I-IV) using our criteria offers novel biomarkers to monitor a variety of physiological and pathological conditions associated with exposure to hypoxia and during recovery. While methods such as pulse oximetry or blood gas analysis are accurate and useful for detecting hypoxia as it occurs, they present limited utility in terms of being able to report prior occurrence of hypoxia or indeed recovery from prior hypoxic exposure. In contrast to using increased hematocrit levels as a marker for prior hypoxia exposure, the suggested gene-based biomarkers may be able to distinguish between chronic hypoxia and recovery from hypoxia with greater accuracy and independent of clinical states that often accompany hypoxia (e.g. volume dysregulation) which may falsely alter hematocrit levels. We believe that the novel biomarkers described here may be used to detect prior hypoxic exposure of up to 2 weeks duration either in isolation or as a combination (e.g. from Categories II & IV) analogous to the manner in which HbA1c is used to report the average blood glucose level over the previous month rather than just at the time of the test while managing patients with Diabetes. We believe that these potential novel biomarkers will be of great benefit to the research community and clinicians as it enables the detection of hypoxia in a post hypoxic state. However, we think it will be important to test the biomarkers identified here in a variety of animal models and clinical settings prior to their utilization.

## Supporting Information

Table S1 **Differential gene expression in both CH and REC conditions.**
(XLS)

Table S2 **Significantly enriched clusters using DAVID.**
(XLS)

Table S3 **Differentially expressed genes in common with Gharib SA et al., Sleep. 2010 Nov;33(11):1439–46.** Common genes expressed in our study after chronic hypoxia exposure and Gharib et al. studying bone marrow of mice exposed to intermittent hypoxia.
(XLS)

## References

[1] Windsor JS, Rodway GW (2007). Heights and haematology: the story of haemoglobin at altitude.. Postgrad Med J.

[2] Kellogg RH (1978). “La Pression barometrique”: Paul Bert’s hypoxia theory and its critics.. Respir Physiol.

[3] Jelkmann W (1986). Erythropoietin research, 80 years after the initial studies by Carnot and Deflandre.. Respir Physiol.

[4] Ebert BL, Bunn HF (1999). Regulation of the erythropoietin gene.. Blood.

[5] Miyake T, Kung CK, Goldwasser E (1977). Purification of human erythropoietin.. J Biol Chem.

[6] Fisher JW (2003). Erythropoietin: physiology and pharmacology update.. Exp Biol Med (Maywood).

[7] Wang GL, Semenza GL (1995). Purification and characterization of hypoxia-inducible factor 1.. J Biol Chem.

[8] Wenger RH (2000). Mammalian oxygen sensing, signalling and gene regulation.. J Exp Biol.

[9] Stroka DM, Burkhardt T, Desbaillets I, Wenger RH, Neil DA (2001). HIF-1 is expressed in normoxic tissue and displays an organ-specific regulation under systemic hypoxia.. Faseb J.

[10] Baze MM, Schlauch K, Hayes JP (2010). Gene expression of the liver in response to chronic hypoxia..

[11] Benita Y, Kikuchi H, Smith AD, Zhang MQ, Chung DC (2009). An integrative genomics approach identifies Hypoxia Inducible Factor-1 (HIF-1)-target genes that form the core response to hypoxia.. Nucleic Acids Res.

[12] Rogers HM, Yu X, Wen J, Smith R, Fibach E (2008). Hypoxia alters progression of the erythroid program.. Exp Hematol.

[13] Dennis G, Jr, Sherman BT, Hosack DA, Yang J, Gao W (2003). DAVID: Database for Annotation, Visualization, and Integrated Discovery.. Genome Biol.

[14] Huang da W, Sherman BT, Lempicki RA (2009). Systematic and integrative analysis of large gene lists using DAVID bioinformatics resources.. Nat Protoc.

[15] Houwen B (2002). Blood film preparation and staining procedures.. Clin Lab Med 22: 1–14,.

[16] Gharib SA, Dayyat EA, Khalyfa A, Kim J, Clair HB (2010). Intermittent hypoxia mobilizes bone marrow-derived very small embryonic-like stem cells and activates developmental transcriptional programs in mice.. Sleep.

[17] Balbir A, Lee H, Okumura M, Biswal S, Fitzgerald RS (2007). A search for genes that may confer divergent morphology and function in the carotid body between two strains of mice.. Am J Physiol Lung Cell Mol Physiol.

[18] Gaber T, Haupl T, Sandig G, Tykwinska K, Fangradt M (2009). Adaptation of human CD4+ T cells to pathophysiological hypoxia: a transcriptome analysis.. J Rheumatol.

[19] Hammond EM, Mandell DJ, Salim A, Krieg AJ, Johnson TM (2006). Genome-wide analysis of p53 under hypoxic conditions.. Mol Cell Biol.

[20] Juul SE, Beyer RP, Bammler TK, McPherson RJ, Wilkerson J (2009). Microarray analysis of high-dose recombinant erythropoietin treatment of unilateral brain injury in neonatal mouse hippocampus.. Pediatr Res.

[21] Komor M, Guller S, Baldus CD, de Vos S, Hoelzer D (2005). Transcriptional profiling of human hematopoiesis during in vitro lineage-specific differentiation.. Stem Cells.

[22] Roiniotis J, Dinh H, Masendycz P, Turner A, Elsegood CL (2009). Hypoxia prolongs monocyte/macrophage survival and enhanced glycolysis is associated with their maturation under aerobic conditions.. J Immunol.

[23] Gheorghe CP, Mohan S, Oberg KC, Longo LD (2007). Gene expression patterns in the hypoxic murine placenta: a role in epigenesis?. Reprod Sci.

[24] Lai Y, Brandhorst H, Hossain H, Bierhaus A, Chen C (2009). Activation of NFkappaB dependent apoptotic pathway in pancreatic islet cells by hypoxia.. Islets.

[25] Pfafflin A, Brodbeck K, Heilig CW, Haring HU, Schleicher ED (2006). Increased glucose uptake and metabolism in mesangial cells overexpressing glucose transporter 1 increases interleukin-6 and vascular endothelial growth factor production: role of AP-1 and HIF-1alpha.. Cell Physiol Biochem.

[26] Salim A, Nacamuli RP, Morgan EF, Giaccia AJ, Longaker MT (2004). Transient changes in oxygen tension inhibit osteogenic differentiation and Runx2 expression in osteoblasts.. J Biol Chem.

[27] van den Beucken T, Magagnin MG, Jutten B, Seigneuric R, Lambin P (2011). Translational control is a major contributor to hypoxia induced gene expression.. Radiother Oncol.

[28] Pistilli EE, Bogdanovich S, Mosqueira M, Lachey J, Seehra J (2010). Pretreatment with a soluble activin type IIB receptor/Fc fusion protein improves hypoxia-induced muscle dysfunction.. Am J Physiol Regul Integr Comp Physiol.

[29] Dill RP, Chadan SG, Li C, Parkhouse WS (2001). Aging and glucose transporter plasticity in response to hypobaric hypoxia.. Mech Ageing Dev.

[30] Harik SI, Behmand RA, LaManna JC (1994). Hypoxia increases glucose transport at blood-brain barrier in rats.. J Appl Physiol.

[31] Savourey G, Launay JC, Besnard Y, Guinet A, Bourrilhon C (2004). Control of erythropoiesis after high altitude acclimatization.. Eur J Appl Physiol.

[32] Xia Y, Warshaw JB, Haddad GG (1997). Effect of chronic hypoxia on glucose transporters in heart and skeletal muscle of immature and adult rats.. Am J Physiol.

[33] Forbes WH (1936). Blood sugar and glucose tolerance at high altutides.. Am J Physiol.

[34] Pison CM, Chauvin C, Fontaine E, Catelloni F, Keriel C (1995). Mechanism of gluconeogenesis inhibition in rat hepatocytes isolated after in vivo hypoxia.. Am J Physiol.

[35] Ogawa Y, Masuzaki H, Isse N, Okazaki T, Mori K (1995). Molecular cloning of rat obese cDNA and augmented gene expression in genetically obese Zucker fatty (fa/fa) rats.. J Clin Invest.

[36] Zhang Y, Proenca R, Maffei M, Barone M, Leopold L (1994). Positional cloning of the mouse obese gene and its human homologue.. Nature.

[37] Hoggard N, Hunter L, Duncan JS, Williams LM, Trayhurn P (1997). Leptin and leptin receptor mRNA and protein expression in the murine fetus and placenta.. Proc Natl Acad Sci U S A.

[38] Bado A, Levasseur S, Attoub S, Kermorgant S, Laigneau JP (1998). The stomach is a source of leptin.. Nature.

[39] Wang J, Liu R, Hawkins M, Barzilai N, Rossetti L (1998). A nutrient-sensing pathway regulates leptin gene expression in muscle and fat.. Nature.

[40] Morash B, Li A, Murphy PR, Wilkinson M, Ur E (1999). Leptin gene expression in the brain and pituitary gland.. Endocrinology.

[41] Schwartz MW, Woods SC, Porte D, Jr, Seeley RJ, Baskin DG (2000). Central nervous system control of food intake.. Nature.

[42] Yingzhong Y, Droma Y, Rili G, Kubo K (2006). Regulation of body weight by leptin, with special reference to hypoxia-induced regulation.. Intern Med.

[43] Ahima RS (2000). Leptin and the neuroendocrinology of fasting.. Front Horm Res.

[44] Anubhuti, Arora S (2008). Leptin and its metabolic interactions: an update.. Diabetes Obes Metab.

[45] Friedman JM, Halaas JL (1998). Leptin and the regulation of body weight in mammals.. Nature.

[46] Ahima RS, Flier JS (2000). Leptin.. Annu Rev Physiol.

[47] Ambrosini G, Nath AK, Sierra-Honigmann MR, Flores-Riveros J (2002). Transcriptional activation of the human leptin gene in response to hypoxia. Involvement of hypoxia-inducible factor 1.. J Biol Chem.

[48] Grosfeld A, Andre J, Hauguel-De Mouzon S, Berra E, Pouyssegur J (2002). Hypoxia-inducible factor 1 transactivates the human leptin gene promoter.. J Biol Chem.

[49] Ou LC, Leiter JC (2004). Effects of exposure to a simulated altitude of 5500 m on energy metabolic pathways in rats.. Respir Physiol Neurobiol.

[50] Ou LC (1980). Hypoxia-induced hemoglobinemia: hypoxic threshold and pathogenic mechanism.. Exp Hematol.

[51] Koistinen PO, Rusko H, Irjala K, Rajamaki A, Penttinen K (2000). EPO, red cells, and serum transferrin receptor in continuous and intermittent hypoxia.. Med Sci Sports Exerc.

[52] Hill NS, Petit RD, Gagnon J, Warburton RR, Ou LC (1993). Hematologic responses and the early development of hypoxic pulmonary hypertension in rats.. Respir Physiol.

[53] Febbraio M, Hajjar DP, Silverstein RL (2001). CD36: a class B scavenger receptor involved in angiogenesis, atherosclerosis, inflammation, and lipid metabolism.. J Clin Invest.

[54] Serghides L, Smith TG, Patel SN, Kain KC (2003). CD36 and malaria: friends or foes?. Trends Parasitol.

[55] Telen MJ (2000). Red blood cell surface adhesion molecules: their possible roles in normal human physiology and disease.. Semin Hematol.

[56] Telen MJ (2005). Erythrocyte adhesion receptors: blood group antigens and related molecules.. Transfus Med Rev.

[57] Kwapiszewska G, Wilhelm J, Wolff S, Laumanns I, Koenig IR (2005). Expression profiling of laser-microdissected intrapulmonary arteries in hypoxia-induced pulmonary hypertension.. Respir Res.

[58] Mwaikambo BR, Yang C, Chemtob S, Hardy P (2009). Hypoxia up-regulates CD36 expression and function via hypoxia-inducible factor-1- and phosphatidylinositol 3-kinase-dependent mechanisms.. J Biol Chem.

[59] Gonzalez-Cinca N, Perez de la Ossa P, Carreras J, Climent F (2004). Effects of thyroid hormone and hypoxia on 2,3-bisphosphoglycerate, bisphosphoglycerate synthase and phosphoglycerate mutase in rabbit erythroblasts and reticulocytes in vivo.. Horm Res.

[60] Pascual J, Pfuhl M, Rivas G, Pastore A, Saraste M (1996). The spectrin repeat folds into a three-helix bundle in solution.. FEBS Lett.

[61] Shotton DM, Burke BE, Branton D (1979). The molecular structure of human erythrocyte spectrin. Biophysical and electron microscopic studies.. J Mol Biol.

[62] Speicher DW (1984). Structural and functional features of the alpha-1 domain from human erythrocyte spectrin.. Prog Clin Biol Res.

[63] Yan Y, Winograd E, Viel A, Cronin T, Harrison SC (1993). Crystal structure of the repetitive segments of spectrin.. Science.

[64] Baines AJ (2009). Evolution of spectrin function in cytoskeletal and membrane networks.. Biochem Soc Trans.

[65] Chakrabarti A, Kelkar DA, Chattopadhyay A (2006). Spectrin organization and dynamics: new insights.. Biosci Rep.

[66] Fenner BJ, Scannell M, Prehn JH (2009). Identification of polyubiquitin binding proteins involved in NF-kappaB signaling using protein arrays.. Biochim Biophys Acta.

[67] Kim JW, Tchernyshyov I, Semenza GL, Dang CV (2006). HIF-1-mediated expression of pyruvate dehydrogenase kinase: a metabolic switch required for cellular adaptation to hypoxia.. Cell Metab.

[68] Adams E (1970). Metabolism of proline and of hydroxyproline.. Int Rev Connect Tissue Res.

[69] Yeh GC, Phang JM (1980). The function of pyrroline-5-carboxylate reductase in human erythrocytes.. Biochem Biophys Res Commun.

[70] Mense SM, Sengupta A, Zhou M, Lan C, Bentsman G (2006). Gene expression profiling reveals the profound upregulation of hypoxia-responsive genes in primary human astrocytes.. Physiol Genomics.

